# Bibliometric analysis of global scientific activity on umbilical cord mesenchymal stem cells: a swiftly expanding and shifting focus

**DOI:** 10.1186/s13287-018-0785-5

**Published:** 2018-02-07

**Authors:** Jian Zhao, Guanyu Yu, Mengxi Cai, Xiao Lei, Yanyong Yang, Qijin Wang, Xiao Zhai

**Affiliations:** 1 Department of Orthopedics, Changhai Hospital, Second Military Medical University, Shanghai, China; 2 Graduate Management Unit, Second Military Medical University, Shanghai, China; 3 Department of Colorectal Surgery, Changhai Hospital, Second Military Medical University, Shanghai, China; 4 Department of Radiation Medicine, Faculty of Naval Medicine, Second Military Medical University, Shanghai, 200433 China; 5 Department of Endocrinology, Changhai Hospital, Second Military Medical University, Shanghai, 200433 China

**Keywords:** Bibliometric, Citation, H-index, UC-MSC, Hotspots

## Abstract

Several studies have focused on umbilical cord-derived mesenchymal stem cells (UC-MSCs) due to their potential therapeutic effects in a cluster of diseases. However, there has been no bibliometric analysis evaluating the evolution in UC-MSC research. Therefore, this study aimed to assess scientific activity regarding UC-MSC research. Publications on UC-MSCs were retrieved from the Science Citation Index-Expanded (SCI-E) of the Web of Science (WoS) from 1975 to 2017. Statistical analyses were performed using Excel, GraphPad Prism 5, and VOSviewer software. Comparative analyses were employed to assess contributions between different countries, institutes, and researchers. With 21.26 citations per paper, 1206 papers cited 25,517 times were included. Mainland China contributed the most with 558 papers, with the most citations (6858 times) and the highest H-index (43). South Korea ranked first for number of papers per million people and per trillion gross domestic product (GDP). Keywords were stratified into two clusters by VOSviewer software: cluster 1, “treatments and effects”; and cluster 2, “characteristics”. The average appearing years (AAY) of keywords in cluster 1 was more recent than that in cluster 2. For promising hotspots, “TNF-α” showed the latest AAY at 2014.09, followed by “migration”, “angiogenesis”, and “apoptosis”. We conclude that the number of publications has been continuously growing dramatically since 2002 and that mainland China and South Korea are the most productive regions. The focus gradually shifts from “characteristics” to “treatments and effects”. Attention should be drawn to the latest hotspots, such as “TNF-α”, “migration”, “angiogenesis”, and “apoptosis”. Furthermore, funding agencies might increase investments in exploring the therapeutic potential of UC-MSCs.

**Electronic supplementary material:**

The online version of this article (10.1186/s13287-018-0785-5) contains supplementary material, which is available to authorized users.

## Background

Mesenchymal stem cells (MSCs) have a significant capacity for self-renewal and differentiation. Activation of MSCs may provide avenues for regenerative medicine due to convenient isolation techniques and immune allorecognition escape [1]. MSCs encompass multipotent cells derived from bone marrow tissue, umbilical cord, and adipose tissue [2]. Comparatively, umbilical cord-derived MSCs (UC-MSCs) are inexhaustible and can be harvested without any invasive medical operations [1]. In addition, UC-MSCs have been shown to have more efficient proliferation with lower immunogenicity than that of bone marrow-derived MSCs [3–5]. As a result, a growing number of studies have reported that UC-MSCs may be an alternative source of stem cells with promising therapeutic effects [6, 7]. Research evidence proposes that UC-MSCs can potentially be used to improve cardiac regeneration [8–11], alleviate cutaneous wounds [1, 12, 13], treat various neurological disorders [3, 14, 15], and ameliorate insulin resistance in type 2 diabetes [16].

However, no bibliometric reports assessing the relevant scientific output and research trends regarding UC-MSCs have been performed. Bibliometrics can assess not only the quantity, but also the quality of publications regarding a specific field, issue, institute, or region [17]. To a certain degree, they can provide details on the development processes in a specific field [18] and can systematically estimate the research activity trends. Moreover, bibliometric analysis can provide reference proposals during government policy establishment, particularly for determining funding-orientation guidance. Until now, bibliometric analysis has been employed for estimating the research trends on dozens of fields such as intracranial aneurysms [19], retina regeneration [20], obesity [21], and spinal tuberculosis [22].

This study aimed to estimate the publication pattern of UC-MSCs worldwide. Data were obtained from the Web of Science (WoS). We systematically assessed publication distribution stratified by geography, institutions, funding agencies, journals, and more. Furthermore, we also assessed the frequency of keywords, and then employed bibliometric mapping to describe the development of UC-MSC research.

## Materials and methods

### Bibliometric data and search strategy

A comprehensive bibliographic retrieval was performed online using the Web of Science (WoS) on 1 July 2017. This was performed on a single day to avoid daily updating bias since the database is still open.

Search keywords were referred to MESH terms from PubMed, and then the search term was used as follows: TI = *UCMSC* OR TI = *UC-MSC* OR (((TI = (umbilical cord) OR TI = (Wharton’s jelly)) AND (TI = (mesenchymal stromal cell*) OR TI = (mesenchymal stem cell*))) AND Language = English. The time period of article publication was from 1 January 1975 to 1 July 2017. For manuscript types, only peer-reviewed articles and reviews were included.

Distributed details such as original countries, institutes, journals, and funding agencies were refined by WoS.

### Data collection

The original data download from WoS were firstly imported into Microsoft Excel 2010, and then were verified and then assessed by two independent researchers (XZ and JZ), respectively. Any difference was unified through discussion. Finally, the following bibliometric parameters were extracted: the quantity of papers, the number of citations, and H-index [23] (https://en.wikipedia.org/wiki/H-index).

Given the differences in aggregate economic volume and populations among countries, we calculated the publication number per million people and per trillion gross domestic product (GDP). The latest information on population and GDP was obtained from the World Bank [24] and the Central Intelligence Agency [25].

### Statistical methods

The time trend of the number of publications was analyzed using a mathematical fitting curve via GraphPad Prism 5 (GraphPad Software Inc., CA, USA). The logistic growth model $\mathrm{f}\left(\mathrm{x}\right)=\mathrm{c}/\left[1+\mathrm{a}\times {\mathrm{e}}^{\left(-\mathrm{b}\times \Big(\mathrm{x}-2001\right)}\right]$ was used to model the cumulative volume of documentation due to its good fitness and ability to predict the future trends in the literature [19, 26], where x represents the year and f(x) is the cumulative volume of papers by year. The year 2001 was defined as year zero since publications were recorded starting from 2002. The inflection point of the logistic growth curve is the point in time when the publication growth rate shifted from positive to negative, and was generated by the formula T = 2001 + ln a/b [19].

The java program VOSviewer (Leiden University, Leiden, Netherlands) was used for mapping and clustering of keywords [27]. It portrayed keywords by colors and sizes of the circles [28] according to keyword occurrences in both titles and abstracts. In addition, hotspots are defined as the keywords of popular scientific fields and their frequency was calculated using VOSviewer.

## Results

### Evaluation of global publications

**Fig. 1:**
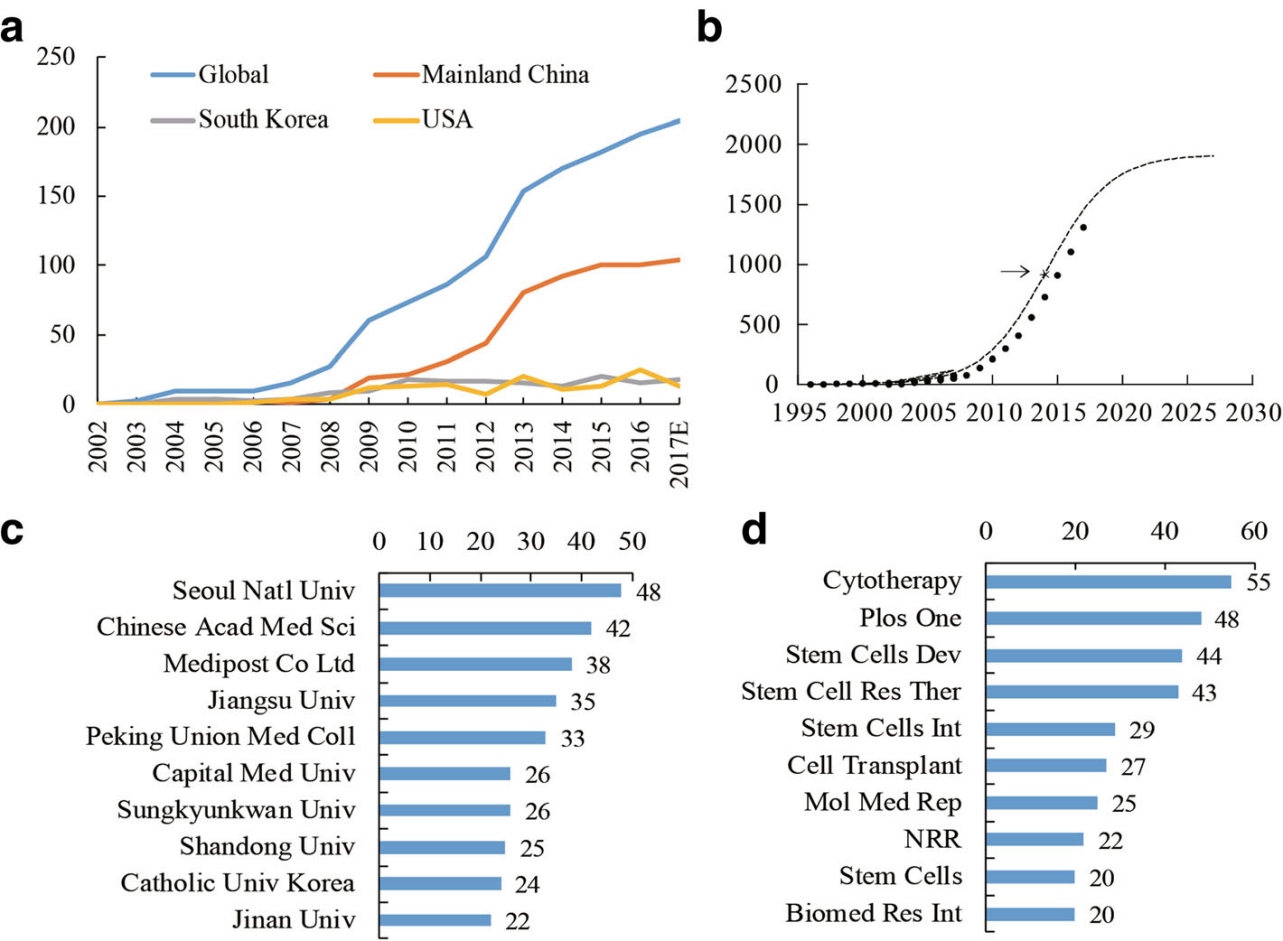
Contributive characteristics of UC-MCS research. **a** The total publications worldwide and the three most productive countries in UC-MCS research. **b** Model fitting curves of growth trends of accumulated number of publications on UC-MCS worldwide. **c** The number of publications on UC-MCS research from the top 10 contribution institutes. **d** The number of publications of the top 10 popular journals on UC-MCS research

We retrieved 1630 publications in total and only included 1206 papers in the analysis (Additional file 1). Figure 1b illustrates that the number of global publications per year increased significantly from 2002. Figure 1b also shows the model fitting curves $\mathrm{f}\left(\mathrm{x}\right)=23523.68/\left[1+695518.94\times {\mathrm{e}}^{\left(-0.33\times \Big(\mathrm{x}-2001\right)}\right]$ of the cumulative number of publications on UC-MSCs. The global inflection point (the point in time when the publication growth rate moved from positive to negative) was calculated to have occurred in 2014.

In regards to the research strength of countries (Table 1), mainland China (558 papers, 46.27%) was the most productive, followed by South Korea (160 papers, 13.27%) and the United States (136 papers, 11.28%). When adjusted by population, South Korea was on top with 3.14 papers per million people. After the adjustment by GDP, South Korea also ranked the first with 82.94 papers per trillion GDP, followed by Iran with 36.33 papers per trillion GDP.

**Table 1:** Publications in the 10 most productive countries

Country	*N*	%	*N* per million people	*N* per trillion GDP	H-index	Total citations
Mainland China	558	46.27	0.41	26.23	43	6858
South Korea	160	13.27	3.14	82.94	36	3838
USA	136	11.28	0.42	7.33	34	3949
Iran	53	4.39	0.64	36.33	9	289
India	43	3.57	0.03	4.93	15	684
Italy	42	3.48	0.68	18.91	17	1029
Taiwan	38	3.15	1.62	33.72	19	2841
Germany	37	3.07	0.46	9.30	16	3395
England	35	2.90	0.54	12.55	14	1056
Japan	29	2.40	0.23	5.88	14	711

*GDP* gross domestic product

### Citation frequency and H-index analysis

Based on WoS database analysis, all publications related to UC-MSCs have been cited 25,517 times with 21.26 citations per paper. In addition, the top 100 UC-MSC research papers contributed 14,252 citations (55.85% of 25,517; Additional file 2). When stratified by region, mainland China was cited the most (6858 times) and achieved the highest H-index (43; Table 1).

### Distribution of highly contributive institutes, journals, and funding agencies

The top 10 contributive institutes are listed in Fig. 1c. Seoul National University in South Korea contributed the most publications with 48 papers published, followed by the Chinese Academy of Medical Sciences (42 publications), and Medipost Co. Ltd., from South Korea (38 publications). Among the top 10 productive institutions, six of them were from China, and the other four were Korean institutes.

There were 333 articles published in the top 10 journals (28.51% of all publications; Fig. 1d). *Cytotherapy* ranked the first (55 articles), followed by *PLoS One* (48 articles) and *Stem Cells and Development* (44 articles).

The top 10 funding bodies are shown in Table 2, with seven funding agencies based in China. The National Natural Science Foundation of China endorsed 255 studies in this field (ranked first, 21.14%), followed by the Jiangsu Province for Outstanding Sci Tech Innovation Team in China (16 studies, 1.33%) and the China Postdoctoral Science Foundation (16 studies, 1.33%).

**Table 2:** The top 10 related funding agencies

Funding agency	*N*	%
National Natural Science Foundation of China	255	21.14%
Jiangsu Province for Outstanding Sci Tech Innovation Team	16	1.33%
China Postdoctoral Science Foundation	16	1.33%
Fundamental Research Funds for the Central Universities	13	1.08%
Natural Science Foundation of Jiangsu Province	12	1.00%
National Institutes of Health (NIH)	12	1.00%
National Research Foundation of Korea (NRF)	12	1.00%
Ministry of Education Science and Technology	11	0.91%
National Basic Research Program of China	10	0.83%
Ministry of Health And Welfare Republic of Korea	9	0.75%

### Highly contributive authors on UC-MSC

The 10 most productive authors contributed 256 papers (21.23%) on UC-MSC research. W. Oh from Medipost Co. Ltd., Biomedical Research Institution, South Korea, contributed the most articles with 31 papers, followed by W.R. Xu from the School of Medicine, Jiangsu University, China, and Z.C. Han from the Chinese Academy of Medical Sciences, Peking Union Medical College, China, with 30 publications each (Table 3).

**Table 3:** The top 10 authors with the most publications related to UC-MSC research

Author	*N*	Total citations	H-index	Country	Affiliation
Oh W	31	1187	20	South Korea	Medipost Co. Ltd., Biomedical Research Institute
Han ZC	30	1014	15	China	Chinese Academy of Medical Sciences, Peking Union Medical College
Xu WR	30	943	13	China	School of Medicine, Jiangsu University
Choi SJ	27	754	14	South Korea	Medipost Co. Ltd., Biomedical Research Institute
Qian H	26	756	12	China	School of Medicine, Jiangsu University
Yang YS	26	871	17	South Korea	Medipost Co. Ltd., Biomedical Research Institute
Zhang X	22	555	11	China	School of Medicine, Jiangsu University
Zhu W	22	752	12	China	School of Medicine, Jiangsu University
Kang KS	21	441	13	South Korea	Seoul National University, College of Veterinary Medicine
Yan YM	21	665	10	China	School of Medicine, Jiangsu University

*UC-MSC* umbilical cord-derived mesenchymal stem cell

### Characteristics of the top 10 UC-MSC articles

When it came to the top 10 most cited articles, there were 7398 citations (28.99%; Table 4). The study by Kern et al. [29], published in 2006, was the most cited article (1382 times) with an average citation of 115.17 per year. Among the 10 most cited articles, five were published in *Stem Cells*, two in *Experimental Hematology*, one in *Blood*, one in the *British Journal of Haematology*, and one in *Haematologica*.

**Table 4:** The top 10 UC-MSC research papers with the most citation frequency

Title	First author	Journal	Year	Citations	Citation frequency per year	Main conclusion
Comparative analysis of mesenchymal stem cells from bone marrow, umbilical cord blood, or adipose tissue	Kern	Stem Cells	2006	1382	115.17	They compared MSCs derived from umbilical cord blood, bone marrow, and adipose tissue regarding morphology, the success rate of isolating MSCs, colony frequency, expansion potential, multiple differentiation capacity, and immune phenotype
Isolation of multipotent mesenchymal stem cells from umbilical cord blood	Lee	Blood	2004	819	58.5	They reported a novel method to obtain single cell-derived, clonally expanded MSCs that are of multilineage differentiation potential
Mesenchymal stem cells in the Wharton’s jelly of the human umbilical cord	Wang	Stem Cells	2004	665	47.5	UC-MSC express matrix receptors (CD44, CD105) and integrin markers (CD29, CD51), and can differentiate into cardiomyocytes
Comparative characteristics of mesenchymal stem cells from human bone marrow, adipose tissue, and umbilical cord blood	Wagner	Experimental Hematology	2005	660	50.77	They provided a foundation for a more reproducible and reliable quality control using genotypic analysis for defining MSCs
Critical parameters for the isolation of mesenchymal stem cells from umbilical cord blood	Bieback	Stem Cells	2004	510	36.43	MSC-like cells can be isolated at high efficacy from full-term UC donations; we regard UC as an additional stem cell source for experimental and potentially clinical purposes
Searching for alternative sources of postnatal human mesenchymal stem cells: Candidate MSC-like cells from umbilical cord	Romanov	Stem Cells	2003	505	33.67	UC vasculature contains a high number of MSC-like elements forming colonies of fibroblastoid cells that may be successfully expanded in culture
Comparison of proliferative and multilineage differentiation potential of human mesenchymal stem cells derived from umbilical cord and bone marrow	Baksh	Stem Cells	2007	457	41.55	They compared HUCPVCs to the “gold standard” bone marrow mesenchymal stromal cells (BMSCs) with respect to their proliferation, differentiation, and transfection capacities
Adult bone marrow is a rich source of human mesenchymal ‘stem’ cells but umbilical cord and mobilized adult blood are not	Wexler	British Journal of Haematology	2003	384	25.6	Adult BM is a reliable source of functional cultured MSCs, but cord blood and peripheral blood stem cell collections are not
Isolation and characterization of human umbilical cord mesenchymal stem cells with hematopoiesis-supportive function and other potentials	Lu	Haematologica	2006	381	31.75	They established a protocol to isolate abundant MSCs from human umbilical cords with a 100% success rate. The comparative study indicates that UC is an excellent alternative to BM as a source of MSCs for cell therapies.
Mesenchymal stem cells promote engraftment of human umbilical cord blood-derived CD34(+) cells in NOD/SCID mice	Noort	Experimental Hematology	2002	315	19.69	Upon co-transplantation, MSCs, but not irradiated CD34^−^ or B cells, promote engraftment of UCB CD34^+^ cells in bone marrow, spleen, and blood.

*BM* bone marrow, *HUCPVC* human umbilical cord perivascular cell, *MSC* mesenchymal stem cell, *UC* umbilical cord, *UCB* umbilical cord blood

### Hotspot analysis

**Fig. 2:**
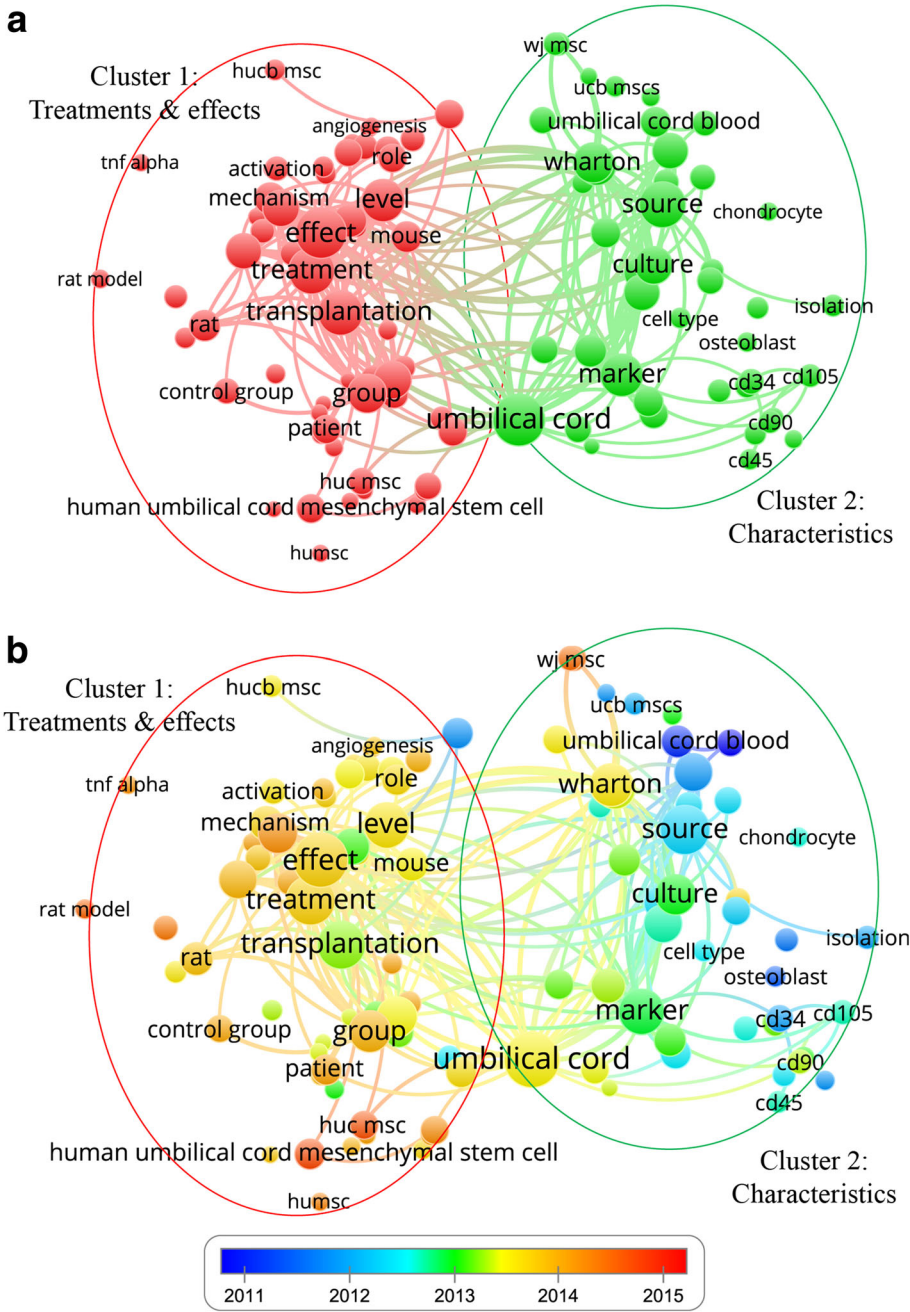
The mapping on keywords of UC-MCS. **a** The keywords were divided into two clusters: cluster 1, “treatments and effects”; and cluster 2, “characteristics”. In general, the smaller the distance between two terms, the larger the number of co-occurrences of the terms. The size of the circle represents the frequency of the keyword, with a larger circle indicating the keyword appears more frequently. **b** Based on the average time of appearance, keywords in blue presented earlier than those in yellow and red

Keywords were extracted from titles and abstracts of 1206 studies and analyzed by VOSviewer software. Keywords appearing more than 100 times were included in the map (Fig. 2 and Additional file 2) and were stratified into two clusters: cluster 1 (treatments and effects; Fig. 2a, left, in red), and cluster 2 (characteristics; Fig. 2a, right, in green). The most frequent keywords in cluster 1 were “effect” (447 times), “treatment” (357 times), and “transplantation” (343 times). In cluster 2, the most frequent keywords were “umbilical cord” (468 times), “marker” (309 times), and “Wharton” (277 times).

Based on the different average appearing year (AAY) of keywords, VOSviewer marked keywords included in the map with different colors (Fig. 2b). Keywords in blue appeared earlier than those in yellow and red. Keywords in the “treatments and effects”-related cluster 1 appeared in more recent years than those in the “characteristics”-related cluster 2. Besides UC-MSC-related keywords, “TNF-α” showed the latest AAY of 2014.09, followed by “migration” with an AAY of 2013.85, “angiogenesis” with an AAY of 2013.78, and “apoptosis” with an AAY of 2013.76 (Additional file 3).

## Discussion

In the present study, we found that the number of global publications per year increased dramatically from 2002, and that the global inflection point may have occurred in 2014. A total of 1206 papers on UC-MSC research have been cited 21.26 times per paper. Mainland China was the most productive region accounting for 46.27% of papers published, with the most citations (6858 times) and the highest H-index (43). South Korea ranked the first regarding number of papers per million people and per trillion GDP. Keywords extracted from titles and abstracts were stratified by VOSviewer software into two clusters, the relatively outdated “characteristics” cluster and the relatively new “treatments and effects” cluster. Furthermore, “TNF-α”, “migration”, “angiogenesis”, and “apoptosis” may be the latest, promising research hotspots in this field.

Prior to 2007, global publications on UC-MSC research exhibited a steady growth, whereas from 2008 onwards, a dramatic growth was witnessed. The regression growth model of cumulative amounts showed an inflection point in 2014; however, there is still a possibility that increasing trends go on longer than expected by the proposed model since function and therapeutic application of UC-MSCs might attract more research attention. Moreover, the scientific communities may pay more attention to these latest issues on UC-MSCs.

When it came to analysis by country, China and South Korea were the most productive countries in this field. We found that both China and South Korea had an overwhelmingly higher number of publications that were supported by local funding agencies and from native innovative institutions than that of other countries. In addition, on the list of top 10 researchers, six were from mainland China and the other four were from South Korea. Oh from South Korea as well as Xu and Han from China contributed the most publications and were leaders in this field. As a result, it is theorized that further publications from these regions may still have an ongoing vital role. Additionally, top scholars from the top institutes can be good choices for partnerships and may also have the priority for more investments and grants.

Regarding journals, those listed in Fig. 2d, such as *Cytotherapy*, *Stem Cells and Development*, *Stem Cell Research & Therapy*, *Stem Cells International*, *Stem Cells*, and *Cell Transplantation*, may be the core journals of UC-MSC research publication. Further studies can be guided for submission to these journals. Subsequently, researchers may pay more attention to research published by the aforementioned journals.

Bibliometrics combined with visualized mapping has been recognized as an effective means of assessing scientific research trends regarding a specific field, both quantitatively and systematically [30]; they can also uncover directions of scientific research, as shown previously [22, 31]. In this study, a gradual shift in terms of research focus from “characteristics” to “treatments and effects” was seen, which is in accordance with the rule of the development of the translational medicine; therefore, the scientific community appears to be interested in the therapeutic potential of UC-MSC research at present. For instance, a pilot clinical trial demonstrated that intravenous transfusion of UC-MSCs was safe and well tolerated, effectively alleviates blood glucose, and increases generation of C-peptide levels and regulatory T cells in a subgroup of type 2 diabetes mellitus patients [32]. Subsequently, those funding agencies may increase investments for these kinds of studies. To illustrate the importance of bibliometric studies, we can also find examples of its impacts in other scientific and professional communities, such as in antimicrobial resistance surveillance. Since data from major surveillance studies are not available for the whole scientific community and are limited by time and region, scholars used bibliometrics methods to compare scientometric results with data from the major surveillance network data and found that bibliometrics provided a fast and reliable global overview of a specific antimicrobial resistance [33]. As a result, bibliometric studies may provide meaningful references for research communities.

While investigating the details provided by visualized mapping, an article titled “Conversion of human umbilical cord mesenchymal stem cells in Wharton's jelly to dopaminergic neurons in vitro: potential therapeutic application for Parkinsonism” in the more recent “treatments or effects” cluster was found to be the most cited article for a total of 251 times at 20.92 citations per year (published in *Stem Cells* in 2006) [34]. This paper proposed that UC-MSCs can be a potential therapeutic strategy for Parkinson’s disease.

Furthermore, our data show that the latest research hotspots are “TNF-α” [35, 36], “migration” [37–41], “angiogenesis” [8, 42–48], and “apoptosis” [49–52]. Therefore, this infers that scientific breakthroughs regarding these hotspots may be achieved in the near future. Furthermore, it pinpoints promising research directions which is of interest to scientists and funding agencies.

### Strengths and limitations

This bibliometric analysis coupled with visualized mapping can provide systematical information on UC-MSC-related research and help readers learn about the evolution of UC-MSC research with relative ease. Furthermore, since publications were assessed based on countries, institutes, and researchers, the analysis can provide relevant information for scientists and funding agencies by highlighting potential cooperative partnerships and providing investment guidance.

However, there are several limitations to this study. First, it may have missed some important research published in other languages since only English papers were retrieved and included in the analysis. Second, all studies could not be identified using one database search. Journals included in the database of Science Citation Index-Expanded (SCI-E) in WoS are described as the world’s leading journals due to a rigorous selection process, and the concept of SCI was based on Bradford’s law in bibliometrics, which can be applied to define a core set of journals or publications. As a result, publications included in WoS may represent studies in the discipline, and WoS provided metadata with further distribution refinement. Third, there exists a discrepancy between bibliometric analysis results and real research conditions; this is due to the database remaining open as it continuously updates studies. Moreover, the increasing trend in the number of papers published might be sustained longer than that calculated by the mathematical model.

## Conclusion

The number of publications regarding UC-MSCs has been continuously growing since 2002. Mainland China and South Korea were found to be the most productive regions. Keyword focus gradually shifted from “characteristics” to “treatments and effects”, meaning that those funding agencies may increase investments for exploring the therapeutic potential of UC-MSCs. It is recommended to pay closer attention to the latest promising hotspots, such as “TNF-α”, “migration”, “angiogenesis”, and “apoptosis”.

## Additional files

Additional file 1: The inclusion and exclusion process of UC-MCS research. (JPG 740 kb)

Additional file 2: Details of all 1206 UC-MSC research papers. (XLSX 273 kb)

Additional file 3: Details of group items by cluster in VOSviewer. (DOCX 21 kb)

## Abbreviations

AAY: Average appearing years
GDP: Gross domestic product
MSC: Mesenchymal stem cell
SCI-E: Science Citation Index-Expanded
UC-MSC: Umbilical cord-derived mesenchymal stem cell
WoS: Web of Science
