# Supplementary figures and images for: Bibliometric analysis of global scientific activity on umbilical cord mesenchymal stem cells: a swiftly expanding and shifting focus
Source: Stem Cell Res Ther. 2018 Feb 7;9:32. doi: 10.1186/s13287-018-0785-5 (PMC5803908; doi:10.1186/s13287-018-0785-5)

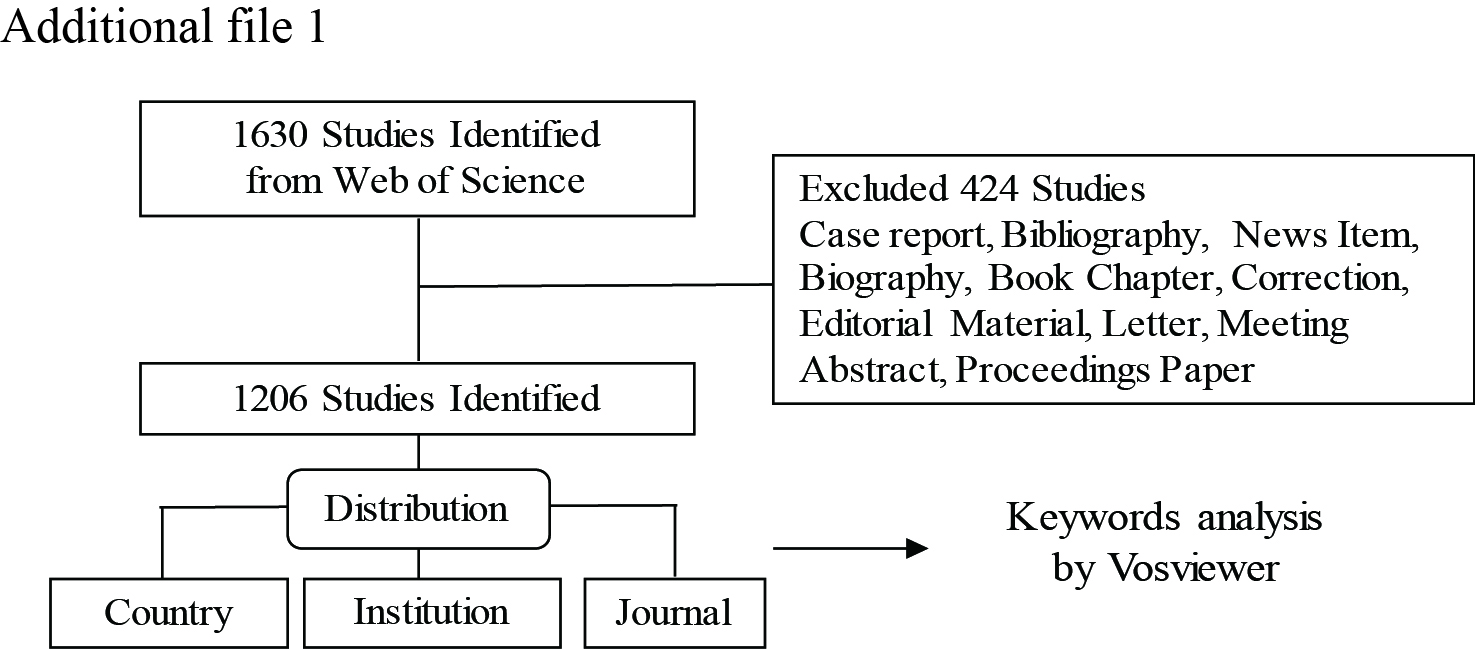

Supplement: Supplementary file 1 — The inclusion and exclusion process of UC-MCS research. (JPG 740 kb) [file 13287_2018_785_MOESM1_ESM.jpg]
