# Supplementary material for: Bibliometric analysis of global scientific activity on umbilical cord mesenchymal stem cells: a swiftly expanding and shifting focus
Source: Stem Cell Res Ther. 2018 Feb 7;9:32. doi: 10.1186/s13287-018-0785-5 (PMC5803908; doi:10.1186/s13287-018-0785-5)
Supplement: Supplementary file 3 — Details of group items by cluster in VOSviewer. (DOCX 21 kb) [file 13287_2018_785_MOESM3_ESM.docx]

Additional file 3. Details of Group Items by Cluster in VOSviewer

|  | label | Occurrences | cluster | Avg. pub. year |
| --- | --- | --- | --- | --- |
| 1 | activation | 96 | 1 | 2013.573 |
| 2 | adipocyte | 76 | 2 | 2011.763 |
| 3 | angiogenesis | 49 | 1 | 2013.776 |
| 4 | animal | 69 | 1 | 2013.536 |
| 5 | apoptosis | 100 | 1 | 2013.76 |
| 6 | application | 156 | 2 | 2013.16 |
| 7 | background aim | 49 | 1 | 2013.898 |
| 8 | beta | 105 | 1 | 2013.552 |
| 9 | bm mscs | 56 | 2 | 2013 |
| 10 | bone marrow | 213 | 2 | 2011.953 |
| 11 | brain | 64 | 1 | 2013.281 |
| 12 | capacity | 215 | 2 | 2012.73 |
| 13 | cd105 | 93 | 2 | 2012.71 |
| 14 | cd29 | 57 | 2 | 2011.947 |
| 15 | cd34 | 106 | 2 | 2012 |
| 16 | cd44 | 77 | 2 | 2012.494 |
| 17 | cd45 | 69 | 2 | 2012.71 |
| 18 | cd73 | 71 | 2 | 2013.028 |
| 19 | cd90 | 88 | 2 | 2013.284 |
| 20 | cell type | 74 | 2 | 2012.554 |
| 21 | change | 114 | 1 | 2013.491 |
| 22 | characteristic | 151 | 2 | 2013.06 |
| 23 | chondrocyte | 56 | 2 | 2012.661 |
| 24 | clinical application | 104 | 2 | 2013.115 |
| 25 | condition | 180 | 2 | 2012.417 |
| 26 | control | 128 | 1 | 2013.188 |
| 27 | control group | 105 | 1 | 2013.943 |
| 28 | culture | 249 | 2 | 2012.972 |
| 29 | cytokine | 116 | 1 | 2013.474 |
| 30 | difference | 129 | 2 | 2013.147 |
| 31 | differentiation potential | 118 | 2 | 2012.331 |
| 32 | disease | 243 | 1 | 2013.416 |
| 33 | effect | 447 | 1 | 2013.673 |
| 34 | efficacy | 95 | 1 | 2013.947 |
| 35 | elisa | 51 | 1 | 2013.98 |
| 36 | expansion | 111 | 2 | 2012.414 |
| 37 | expression level | 66 | 1 | 2013.97 |
| 38 | flow cytometry | 120 | 2 | 2013.383 |
| 39 | function | 225 | 1 | 2013.844 |
| 40 | group | 270 | 1 | 2013.859 |
| 41 | huc msc | 117 | 1 | 2014.487 |
| 42 | hucb msc | 70 | 1 | 2013.486 |
| 43 | hucmsc | 117 | 1 | 2014.12 |
| 44 | human umbilical cord blood | 137 | 1 | 2011.92 |
| 45 | human umbilical cord mesenchymal stem cell | 139 | 1 | 2014.54 |
| 46 | humsc | 48 | 1 | 2014.167 |
| 47 | humscs | 43 | 1 | 2013.744 |
| 48 | immunohistochemistry | 53 | 1 | 2013.019 |
| 49 | important role | 43 | 1 | 2013.349 |
| 50 | improvement | 75 | 1 | 2013.52 |
| 51 | isolation | 79 | 2 | 2012.025 |
| 52 | jelly | 246 | 2 | 2013.342 |
| 53 | level | 311 | 1 | 2013.556 |
| 54 | liver | 59 | 1 | 2013.848 |
| 55 | marker | 309 | 2 | 2012.926 |
| 56 | mechanism | 180 | 1 | 2013.661 |
| 57 | mesenchymal stromal cell | 123 | 2 | 2013.48 |
| 58 | migration | 68 | 1 | 2013.853 |
| 59 | model | 235 | 1 | 2014.094 |
| 60 | month | 63 | 1 | 2013.937 |
| 61 | morphology | 136 | 2 | 2012.537 |
| 62 | mouse | 166 | 1 | 2013.452 |
| 63 | neuron | 90 | 1 | 2012.522 |
| 64 | neurotrophic factor | 43 | 1 | 2013.349 |
| 65 | osteoblast | 61 | 2 | 2011.557 |
| 66 | passage | 98 | 2 | 2013.174 |
| 67 | pathway | 95 | 1 | 2013.874 |
| 68 | patient | 141 | 1 | 2013.972 |
| 69 | phenotype | 105 | 2 | 2012.429 |
| 70 | rat | 165 | 1 | 2013.77 |
| 71 | rat model | 57 | 1 | 2014.368 |
| 72 | recovery | 93 | 1 | 2013.129 |
| 73 | regenerative medicine | 87 | 2 | 2013.644 |
| 74 | regulation | 62 | 1 | 2013.661 |
| 75 | response | 128 | 1 | 2013.414 |
| 76 | role | 135 | 1 | 2013.615 |
| 77 | safety | 47 | 1 | 2013.362 |
| 78 | source | 367 | 2 | 2012.354 |
| 79 | stromal cell | 78 | 2 | 2012.692 |
| 80 | survival | 68 | 1 | 2013.662 |
| 81 | therapeutic effect | 87 | 1 | 2014.241 |
| 82 | tissue engineering | 93 | 2 | 2012.645 |
| 83 | tnf alpha | 46 | 1 | 2014.087 |
| 84 | transplantation | 343 | 1 | 2013.225 |
| 85 | treatment | 357 | 1 | 2013.681 |
| 86 | uc msc | 146 | 1 | 2013.582 |
| 87 | ucb | 109 | 2 | 2010.587 |
| 88 | ucb msc | 48 | 2 | 2011.938 |
| 89 | ucb mscs | 68 | 2 | 2012.088 |
| 90 | ucmsc | 59 | 1 | 2013.627 |
| 91 | ucmscs | 52 | 1 | 2013.962 |
| 92 | umbilical cord | 468 | 2 | 2013.451 |
| 93 | umbilical cord blood | 147 | 2 | 2011.259 |
| 94 | umbilical cord mesenchymal stem cell | 50 | 1 | 2013.86 |
| 95 | use | 176 | 2 | 2013.267 |
| 96 | vivo | 90 | 1 | 2013.933 |
| 97 | week | 192 | 1 | 2013.078 |
| 98 | wharton | 277 | 2 | 2013.527 |
| 99 | wj msc | 90 | 2 | 2014.322 |
| 100 | wj mscs | 87 | 2 | 2014.322 |
